# The TRAPPIST Repair: A Novel TRAnsabdominal PrePeritoneal Intervention for ParaSTomal Hernias – Case Report With Video-Vignette

**DOI:** 10.3389/jaws.2025.15627

**Published:** 2026-01-09

**Authors:** Francesco Brucchi, Pieter Pletinckx, Filip Muysoms

**Affiliations:** 1 Department of Pathophysiology and Transplantation, University of Milan, Milan, Italy; 2 Department of Surgery, Maria Middelares Ghent, Ghent, Belgium

**Keywords:** parastomal hernia, preperitoneal repair, robotic surgery, abdominal wall, stoma

## Abstract

**Introduction:**

Parastomal hernia (PSH) is a common complication following stoma formation, often requiring surgical repair. While techniques such as Sugarbaker and Pauli have improved outcomes, concerns persist regarding intraperitoneal mesh exposure and the disruption of the transversus abdominis muscle insertion during retromuscular repairs. We describe a novel robotic transabdominal preperitoneal Intervention for PSH repair (TRAPPIST), with mesh placement between the peritoneum and posterior rectus sheath, potentially offering anatomical and functional advantages.

**Materials and Methods:**

We report the case of a male patient with symptomatic PSH after left-sided colostomy. Robotic repair was performed using a transabdominal preperitoneal approach. A wide peritoneal flap was created to access the preperitoneal space, followed by lateralization of the stoma, partial closure of the hernia defect, and placement of a lightweight large-pore mesh within the preperitoneal compartment. The mesh was secured with interrupted sutures, and the peritoneum was closed to isolate the prosthesis from the abdominal cavity.

**Results:**

The procedure was completed without complications. The postoperative course was uneventful, with discharge on day 4. At 2 months follow-up, no signs of recurrence, mesh-related complications, or stoma dysfunction were observed.

**Conclusion:**

TRAPPIST repair is technically feasible and may reduce mesh-related complications by avoiding intraperitoneal exposure. However, due to the complexity of wide peritoneal dissection, this technique requires experience and careful patient selection. It can serve as a first-line option with the possibility of conversion to Sugarbaker or Pauli repair if needed. Further studies are warranted to assess long- term outcomes.

## Introduction

Parastomal hernia (PSH) is one of the most common and challenging complications following stoma formation, with reported incidence rates ranging from 30% to 50%, depending on the type of stoma and duration of follow-up [1, 2]. Surgical repair remains the mainstay of treatment for symptomatic cases, but the optimal technique continues to be debated due to high recurrence rates and the risk of mesh-related complications [3, 4]. Moreover, as the local treatment of these hernias requires extensive dissection of the stoma and mesh positioning in close proximity to the colon or small bowel, many surgeons remain hesitant to undertake this type of procedure [5].

Traditional approaches—including local fascial repair, stoma sitting, and mesh reinforcement—have evolved significantly over the past 2 decades. Sugarbaker, Keyhole, Sandwich, and hybrid 3D techniques have demonstrated clear advantages over non-mesh repairs, particularly in terms of lowering recurrence rates and enhancing long-term surgical outcomes [3, 6, 7]. However, intraperitoneal mesh placement could carry risks of bowel erosion, fistula formation, and infection, especially in the context of a contaminated field around the stoma [4, 6].

The Pauli repair, first described in 2016, advanced the field by combining lateralization of the stoma limb with retromuscular mesh placement and closure of the posterior sheath, thereby isolating the mesh from the peritoneal cavity and reducing the risk of mesh-related complications [8]. Subsequent adaptations of the Pauli technique utilizing minimally invasive and robotic platforms have demonstrated promising short-term outcomes, with enhanced visualization and precision facilitating complex dissection and mesh positioning [9]. Nevertheless, all published robotic Pauli repairs to date have described mesh placement in the retromuscular space. Notably, even so-called “preperitoneal” Pauli variants, such as those by Lambrecht et al. [10] and Almoguera González et al. [11], involved retrorectus dissection with partial transversus abdominis release, thus maintaining a retromuscular rather than a true preperitoneal plane.

Here we propose a novel robotic transabdominal preperitoneal intervention for PSH (TRAPPIST repair), with mesh positioned in the preperitoneal plane, which could offer anatomical and functional advantages.

## Case Description

### Patient

**FIGURE 1:**
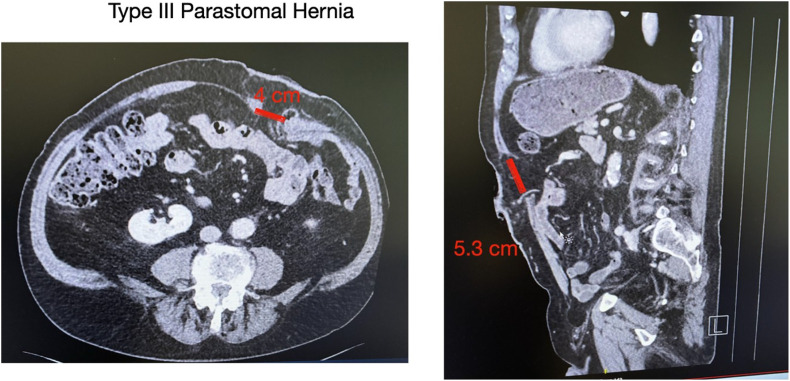
Preoperative CT imaging. Axial (left) and coronal (right) views showing a parastomal hernia measuring 4 cm in width and 5.3 cm in length. According to the European Hernia Society (EHS) classification, this corresponds to a type III parastomal hernia.

A male patient, 83 years old, with a history of rectal cancer (final pathology ypT3N1aM0R0) treated with neoadjuvant chemotherapy, followed by a laparoscopic low anterior resection with permanent end colostomy in October 2024, without adjuvant therapy, presented with a symptomatic PSH, progressively enlarging and causing discomfort. Preoperative CT confirmed a parastomal hernia with a fascial defect of approximately of 4 cm in width and 5.3 cm in length (type III) [12] (Figure 1). Given the clinical presentation and the impact of the parastomal hernia on the patient’s quality of life, surgical indication was established for a robotic parastomal hernia repair with mesh placement.

### Surgical Technique

**FIGURE 2:**
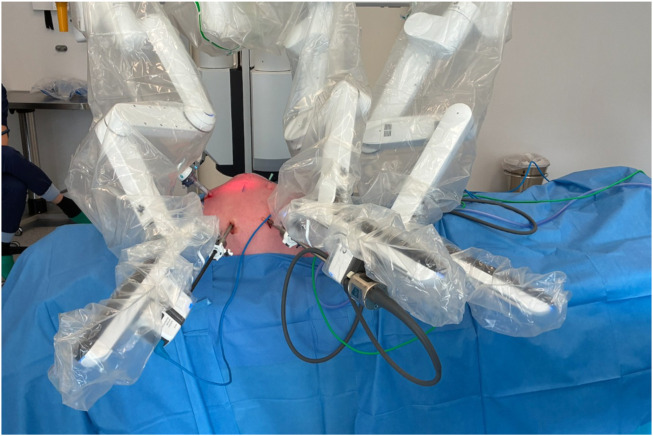
Robotic docking and trocar configuration. The image shows the docking of the robot with the positions of the three 8-mm robotic trocars and the 12-mm laparoscopic trocar used by the bedside assistant.

In the Video available at the following link (https://figshare.com/s/77ffc96478096627ebf6), the surgical technique is demonstrated in detail, illustrating each step of the procedure. The patient was placed supine under general anesthesia. The stoma site was prepared, and an occlusive dressing was applied over the stoma during the procedure to minimize spillage and reduce the risk of wound contamination. Pneumoperitoneum was established using a Veress needle at Palmer’s point, and intra-abdominal pressure was maintained at 12 mmHg. The first 8-mm robotic trocar was placed in the right flank for the camera. Two additional 8-mm robotic ports were positioned under direct vision: one in the right hypochondrium and one in the right iliac fossa, allowing optimal triangulation. A 12-mm assistant trocar was placed in the epigastrium (Figure 2). In selected complex cases, this additional assistant trocar may facilitate stoma mobilization and improve exposure, particularly when dense adhesions are present around the ostomy.

**FIGURE 3:**
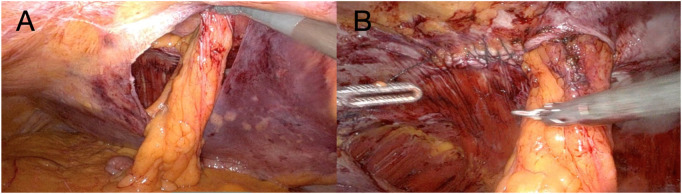
**(A)** The image demonstrates the extent of the peritoneal flap at the completion of the dissection phase; **(B)** The image shows closure of the partial defect performed with a 2-0 absorbable barbed suture.

Initial laparoscopic exploration revealed reducible herniated contents and minimal adhesions. After laparoscopic exploration, the Da Vinci X robotic system was docked. Three robotic arms were utilized throughout the procedure. The camera was positioned on the central robotic arm, with a monopolar scissor on the right arm and a bipolar grasper on the left arm. A peritoneal flap was created from the medial aspect of the stoma, applying principles similar to those used in ventral TAPP hernia repair, providing access to the preperitoneal space (Figure 3A).

**FIGURE 4:**
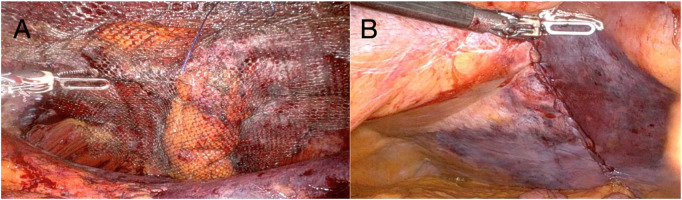
**(A)** Dynamesh®-CICAT (25 × 20 cm) secured with six interrupted 3-0 Vicryl sutures and n- butyl-2-cyanoacrylate (Liquiband Fix8™); **(B)** Final view after dissection. The lateralized stoma is not visible; the peritoneal defect was closed with absorbable 3-0 barbed sutures.

Careful blunt and sharp dissection extended laterally and caudally at least 10 cm cranially and caudally to the ostomy, creating an adequate overlap and preperitoneal space for mesh placement. The hernia sac was incised circumferentially, and the fascial defect was visualized. The hernia defect was then partially closed and tailored to accommodate the passing bowel using a 2-0 absorbable barbed running suture (Figure 3B). The stoma was lateralized as needed to optimize the mesh configuration and secured to the anterior abdominal wall using a 3-0 absorbable barbed running suture. To avoid direct contact between the bowel and the mesh, the visceral loop was deliberately lateralized and anchored to the abdominal wall. This configuration ensures that only the mesocolic surface lies against the mesh. Care was taken to grasp the mesentery without compromising the vascular supply. Finally, the peritoneal defect was partially closed with a running barbed suture around the bowel, proceeding from lateral to medial, without causing any stricture. A DynaMesh®-CICAT (dimensions: 25 × 20 cm) visible large-pore, lightweight mesh was placed, allowing for postoperative MRI visualization of the implant. It was introduced via a 12-mm trocar and positioned within the preperitoneal space, covering the defect with generous overlap. The mesh was secured using 6 interrupted 3/0 Vicryl sutures and n-butyl-2-cyanoacrylate (Liquiband Fix8™, Advanced Medical Solutions plc) (Figure 4A). Peritoneal closure was completed using a running barbed suture, isolating the mesh from intra-abdominal contents (Figure 4B). No drains were placed. The stoma site was inspected for function and vascular integrity.

### Patient Outcome

The postoperative course was uneventful. The patient resumed oral intake on postoperative day 1 and was discharged on postoperative day 4. At 2 months follow-up, no signs of hernia recurrence, mesh- related complications, or stoma dysfunction were observed. The patient was highly satisfied with the cosmetic outcome and the rapid return to daily activities, reporting a significant improvement in overall quality of life.

## Discussion

Parastomal hernia repair remains a technically demanding procedure, with no universally accepted gold standard. Among mesh-based repairs, the Sugarbaker technique remains widely used and is associated with lower recurrence rates than keyhole repairs. However, the intraperitoneal position of the mesh in the Sugarbaker method carries an inherent risk of adhesions, fistulas, and mesh-related visceral complications. The Pauli technique represents a promising alternative with extraperitoneal mesh placement, but it is technically demanding, involving retromuscular dissection and potential disruption of the transversus abdominis muscle insertion, which contributes to its steep learning curve. Early series report morbidity rates as high as 34%, including seroma, surgical site infections, and postoperative ileus. Moreover, long-term data remain limited, with most studies based on small cohorts and short follow-up.

Several recent meta-analyses have evaluated different mesh placement techniques for PSH repair [13]. The Sandwich and Hybrid 3D techniques have shown superior performance in terms of recurrence reduction and cumulative complication rates compared to keyhole and classical Sugarbaker repairs. The Sandwich method, which combines keyhole and Sugarbaker principles with dual mesh placement, demonstrated favorable outcomes in recurrence prevention, but concerns remain regarding higher infection rates (6.4%) potentially related to the use of polypropylene mesh in an intraperitoneal position [13, 14].

The Hybrid 3D approach, utilizing preformed, anatomically contoured meshes in a preperitoneal plane, has been associated with reduced recurrence, fewer complications, and lower surgical site infection rates compared to traditional Sugarbaker repairs [7, 14–16]. Notably, this improved safety profile is likely attributed to the preperitoneal positioning of the mesh, which reduces direct bowel contact, rather than to the mesh material itself [17]. However, despite promising short-term data, the Hybrid 3D technique remains technically demanding, often requiring retroperitoneal dissection, multiple surgical stages, and advanced laparoscopic skills [18].

Overall, although different approaches show specific advantages, no single technique has emerged as superior in all clinical scenarios, and robust, long-term comparative data remain lacking. The preperitoneal approach described in this report combines the principles of anatomical reinforcement with minimal invasiveness. Placement of the mesh between the peritoneum and the posterior rectus sheath avoids direct intraperitoneal mesh contact, potentially reducing adhesions, bowel erosion, and mesh-related complications. The preperitoneal plane also offers enhanced tissue integration and mechanical stability. Interestingly, in 2021 Ayuso et al. described a robotic Sugarbaker repair in which an inferolateral preperitoneal flap was developed to increase mesh overlap [19]. This detail may be considered a conceptual step toward a fully preperitoneal approach, later realized in the TRAPPIST technique, which completely eliminates intraperitoneal mesh contact.

Recently, in abdominal wall surgery, concerns have been raised regarding the placement of retromuscular meshes and the disruption of the retromuscular plane, as this may complicate potential future procedures in the case of recurrence. In 2024, Valenzuela et al. published a study on patients with umbilical hernias and diastasis, employing an endoscopic totally preperitoneal (PeTEP) approach. They accessed the preperitoneal space via the Retzius space, achieving excellent outcomes and contributing to the growing popularity of the preperitoneal technique [20]. The following year, Muñoz-Rodríguez et al. described a cranial approach for the same procedure, further reflecting the increasing scientific interest currently surrounding preperitoneal plane surgery [21]. Compared to the retromuscular Pauli repair, our technique avoids disruption of the posterior rectus sheath and does not require opening the linea alba, preserving abdominal wall integrity and potentially minimizing the impact on core function. The use of a peritoneal flap allows wide lateral dissection without the need for transversus abdominis release (TAR), which is otherwise often necessary in retromuscular repairs.

In contrast to the classical Sugarbaker method, our approach places the mesh entirely within the preperitoneal compartment, with the peritoneum providing an additional protective barrier between the prosthesis and the abdominal viscera. This may reduce the risk of adhesions, mesh erosion, and complications from tacker fixation in proximity to the bowel.

Nevertheless, this technique also presents specific limitations. Dissecting the peritoneum in this anatomical region is not always straightforward and requires significant surgical expertise. Surgeons with prior experience in transabdominal preperitoneal (TAPP) repair of ventral hernias may find this step more intuitive. In patients with a particularly thin or fragile peritoneum, adequate dissection and flap creation may prove challenging, potentially limiting the feasibility of the procedure. Therefore, careful patient selection is critical.

We also believe that this technique could serve as a first-line attempt for PSH repair. If dissection proves technically unmanageable or the anatomical conditions are suboptimal, the procedure allows for conversion to a standard Sugarbaker or retromuscular Pauli repair as a bailout strategy. Robotic assistance facilitates this technically demanding approach, providing superior visualization, dexterity, and access to confined spaces, particularly around the stoma [9]. The lateralization of the bowel loop follows the protective principles of the Sugarbaker technique but avoids exposing the mesh intraperitoneally.

While this approach shows promise, it remains technically challenging and should be reserved for selected patients with favorable anatomy and minimal adhesions. Although we do not exclude the potential applicability of the TRAPPIST technique to EHS type II and IV hernias, its feasibility in cases with large concomitant midline defects may be considerably reduced due to the higher risk of peritoneal tears and technical difficulties in maintaining an intact preperitoneal plane. Further studies with larger cohorts and long-term follow-up are necessary to confirm the safety, efficacy, and recurrence rates of this technique.

## Conclusion

TRAPPIST repair is feasible and may reduce mesh-related complications. However, it requires experience and careful patient selection. Thin peritoneum may limit feasibility, and conversion to Sugarbaker or Pauli remains an option. Further studies are needed to confirm long-term safety, efficacy, and the role of this approach in PSH repair.

## Data Availability

The original contributions presented in the study are included in the article/supplementary material, further inquiries can be directed to the corresponding author.
